# Commentary: Development of magnitude processing in children with developmental dyscalculia: space, time, and number

**DOI:** 10.3389/fpsyg.2015.00804

**Published:** 2015-06-09

**Authors:** Jean-Paul Fischer

**Affiliations:** INTERPSY Laboratory, University of LorraineNancy, France

**Keywords:** developmental dyscalculia, exact number processing, approximate number system, null hypothesis testing, statistical power

Because mathematics is fundamentally symbolic—even totally symbolic at a higher level—the recent finding by Skagerlund and Träff (2014a) that fourth graders with developmental dyscalculia (DD) exhibit intact symbolic number processing seems curious. The importance of symbolism in mathematics is such that the finding seems contradictory with the notion of dyscalculia. Here I discuss three concerns, in recognizing that other aspects of the analysis by Skagerlund and Träff are well controlled.

(1) The finding is based on the acceptance of H_0_ in a study with small power because there were only 19 children with DD. Furthermore, the decision to accept H_0_ was based on a *p* only greater than 0.05 in the Number naming test (NN) and 0.22 in the Symbolic number comparison (SNC). If one uses a directional test for the two-group comparisons (with predicted direction), these *p* can be split in two. Even if the two failures to reject H_0_, in the NN and SNC tests, increase the probability that H_0_ is right, this probability would therefore remain small or medium. Consequently, it is risky to interpret the failures to reject H_0_ as a proof of intact symbolic number processing in DD. Another research by Skagerlund and Träff (2014b) supports this reservation: with children only about 1 year older than in the present research, they found that the children with DD display weaknesses or problems with symbolic number processing.
(2) When one uses a *t*-test for independent samples both the SNC and NN tests show highly significant differences between the DD and the TA4 (fourth graders with typical ability) children: *t*_(47)_ = 2.965, *p* < 0.005 for the SNC test and *t*_(49)_ = 3.185, *p* < 0.003 for the NN test.

These results, computed on the original response times (RT), are not sufficient for determining the factors involved in DD: for example, non-numerical “lexical speed” (assessed with the color naming RAN test) could explain the result of the NN test. Nevertheless, they are important because the a posteriori use of adjusted RT (with ANCOVA) in order to control some factors (such as lexical speed) is questionable. Why is the color naming task a covariate for enumeration and not for subitizing? Why are the two other domain-general abilities not used as covariates?

More generally, analysis of covariance is not an adequate method of analysis when the treatments are applied to nonequivalent groups as are the DD, TA4, and TA2 groups (Huitema, 1980). In ANCOVA the adjusted mean difference of two groups can be interpreted as an unbiased estimate of what the mean difference would be if both groups had exactly the same covariate mean. That is, the adjusted difference can be interpreted as the mean difference that would be expected if a matching design had been employed. When the two groups are non-equivalent on the covariate, however, the adjusted mean difference may be impossible to interpret in this way because a matching procedure cannot be designed. For example, in the color naming task, the DD mean (59.16) was significantly greater than the TA4 mean (44.88): this makes difficult (if not impossible) a matching procedure on this covariate.

Marascuilo and Serlin (1988) tried to prevent this misinterpretation of the use of ANCOVA when they wrote: “One often hears that the analysis of covariance should not be used whenever the covariate means *X*_1_, *X*_2,._.., *X*_*k*_ are essentially equal, since adjustment to a common value accomplishes little, and that it should be used whenever there are large differences in the covariate means for the converse reason. (...) In fact, just the opposite is true.” (p. 611).

(3) The analysis of errors supports the conclusion that the DD group was lower than the TA4 group at these symbolic tests. Notably, for the SNC test, 2 DD children (i.e., 10.5 %) were excluded because they had an error rate of more than 20%, whereas no TA4 child was excluded. Moreover, in spite of these exclusions, the mean error rate of the DD children (5.9%) was greater than the mean error rate of the TA4 children (4.6%). A demonstration in two steps, first claiming that there is no significant difference in error, and, next, that there is no significant difference in RT is not completely satisfactory when DD children have both higher error rates and longer RT than TA4 children. To combine errors and RT, I suggest the use of an error corrected median RT. The correction attributes a RT greater than any other to the erroneous responses (too long outliers are not a problem with median RT, and too short RT can be processed as errors). Such analysis makes the debatable assumption that errors do not result from violating the instructions (respond quickly without making errors), but from the incapacity to follow these instructions. However, in avoiding the exclusion of the two DD children, this analysis would perhaps better reflect the actual capacities of the DD children.

In conclusion, I would emphasize that the question of intact symbolic number processing in DD is of great theoretical importance in developmental psychology. Intact symbolic number processing is a fundamental reason for which Skagerlund and Träff's finding reverses the developmental trajectory and interaction between the two postulated number systems (symbolic exact and approximate) proposed in Noël and Rousselle's (2011) model.

## Conflict of interest statement

The author declares that the research was conducted in the absence of any commercial or financial relationships that could be construed as a potential conflict of interest.
